# Diagnostic Value of AngioPLUS Microvascular Imaging in Thyroid Nodule Diagnosis Using Quantitative and Qualitative Vascularity Grading

**DOI:** 10.3390/biomedicines10071554

**Published:** 2022-06-29

**Authors:** Nonhlanhla Chambara, Shirley Yuk Wah Liu, Xina Lo, Michael Ying

**Affiliations:** 1 Department of Health Technology and Informatics, The Hong Kong Polytechnic University, Hung Hom, Kowloon, Hong Kong, China; nonhlanhla.chambara@connect.polyu.hk; 2 Department of Surgery, The Chinese University of Hong Kong, Prince of Wales Hospital, Shatin, New Territories, Hong Kong, China; liuyw@surgery.cuhk.edu.hk; 3 Department of Surgery, North District Hospital, Sheung Shui, New Territories, Hong Kong, China; dr.xina@gmail.com

**Keywords:** ultrasound, Doppler, TIRADS, malignancy-risk stratification, vascularity, thyroid nodule

## Abstract

This study investigated the diagnostic value of the Angio Planewave Ultrasensitive (AngioPLUS) Doppler ultrasound in improving the efficacy of grey scale ultrasound in thyroid nodule diagnosis. The EU TIRADS was used for the grey scale ultrasound assessment of 94 thyroid nodules. conventional Doppler and AngioPLUS Doppler ultrasound images were evaluated using qualitative vascularity grading, where predominant central vascularity indicated malignancy-suspicion, and quantitative regional vascularity assessment, where predominant peripheral vascularity using a ratio vascularity index (RVI) of >1 indicated benign disease. Diagnostic performance outcomes of sole and combination approaches were calculated based on final pathologic results. Using sole EU TIRADS and AngioPLUS + power Doppler imaging (APDI) based on qualitative vascularity and RVI, the results were a sensitivity of 83.3% vs. 83.3 vs. 66.7% and a specificity of 50% vs. 81.3% vs. 73.4, respectively. EU TIRADS combined with APDI significantly improved the specificity using both qualitative vascularity and RVI assessment approaches (84.4% and 81%, respectively, *p* < 0.05); and slightly reduced the sensitivity (76.7% and 58.1%). For cytologically-equivocal thyroid nodules, the combination approach using qualitative vascularity assessment outperformed the EU TIRADS (sensitivity: both were 88.9%; specificity: 77.4% vs. 38.7%, *p* < 0.05; and AUROC: 0.83 vs. 0.62, *p* < 0.05). APDI combined with EU TIRADS is diagnostically efficient in stratifying thyroid nodules, particularly cytologically-equivocal nodules.

## 1. Introduction

Thyroid cancer is the most prevalent endocrine malignancy globally and the fifth most common malignancy in women accounting for about 5% of all female cancer diagnoses worldwide [1]. The incidence of thyroid cancer has increased over the years with a moderate increase in morbidity with increasing age observed in women and higher mortality rates observed in men due to diagnosis at advanced stages [2,3]. The increase in incidence has mainly been observed in papillary thyroid cancer which accounts for over 85% of thyroid cancers, and this has mostly been attributed to the overdiagnosis of indolent cases of thyroid malignancy [4,5,6].

Thyroid ultrasound is the primary diagnostic imaging modality, which is routinely performed clinically for all patients with suspected or known thyroid neoplasms. Current ultrasound imaging systems have high spatial resolution and excellent temporal resolution, which help confirm the presence of a nodule, demonstrating features with/without suspicion of malignancy and guiding biopsies [7]. The ultrasound diagnosis of thyroid nodules typically involves malignancy risk stratification based on predictive ultrasound features to facilitate triaging nodules for biopsy and the pre-operative staging of thyroid cancer [8,9]. Thyroid Imaging Reporting and Data System (TIRADS) guidelines based on the scoring of multiple suspicious ultrasound features have emerged to improve the diagnostic accuracy of sole features. However, thyroid nodule vascularity assessment is not usually incorporated in the risk stratification categories of some TIRADS which mainly focus on grey scale ultrasound assessment [10].

Increased vascularity or microvascularization are anticipated consequences of abnormal angiogenesis during carcinogenesis as cancer cells invade the nodule areas that are deficient in blood vessels [11,12]. Conventional colour Doppler (or colour flow imaging (CFI) and power Doppler ultrasound (or power Doppler imaging (PDI)) have been commonly used in thyroid nodular vascularity assessment to complement grey scale ultrasound findings. Marked central vascularity, intranodular vascularity, and/or increased chaotic intranodular central vascularity on CFI and PDI have been suggested to be associated with suspicion for malignancy while peripheral vascularity is linked with benignity [13,14,15,16]. However, this assertion remains contested due to variable findings and some studies suggesting that vascularity patterns on CFI and PDI modes have little value in malignancy prediction even when combined with grey scale ultrasound features [17,18,19]. The poor diagnostic performance of conventional Doppler methods for malignancy prediction can also be attributed to the poor sensitivity of these methods in microvascularity pattern assessment.

Recent innovations in microvascular ultrasound imaging techniques with superb detection of microvascular blood flow include superb microvascular imaging (SMI) and Angio Planewave Utrasensitive (AngioPLUS) imaging. Both techniques are purported to outperform CFI and PDI techniques, which have poor sensitivity in depicting microvascular flow, small blood vessel branching, and low blood flow velocity [20,21]. Some thyroid studies conducted with SMI have suggested that intranodular vascular flow with penetrating and more branching vessels is suggestive of malignancy, while dotted linear vascular flow patterns with fewer vessels are suggestive of benign disease [22,23]. Although AngioPLUS has been suggested to be diagnostically effective in differentiating parathyroid lesions from other lesions [24], its role in the malignancy risk assessment of thyroid nodules lacks exploration.

This current study sought to determine the diagnostic value of AngioPLUS in thyroid nodule differentiation based on overall assessment and in cytologically-equivocal nodules. The diagnostic value was evaluated using a qualitative vascularity grading approach and quantitative regional vascularity ratio analysis in combination with the European (EU) TIRADS. PDI coupled with AngioPLUS and combined with EU TIRADS demonstrated improved overall diagnostic efficacy, more so in cytologically-equivocal thyroid nodules.

## 2. Materials and Methods

### 2.1. Study Type

This was a prospective analytical observational study that received ethical approval from The Hong Kong Polytechnic University Institutional Human Subjects Ethics Sub-committee (Registration Number: HSEARS20190123004). Consecutive case analysis and non-probability sampling were applied. Cross-sectional cohorts of patients with thyroid nodules and/or suspicion of thyroid cancer were purposively recruited at the Prince of Wales Hospital Department of Surgery and its affiliates from May 2019 to August 2021. Informed consent was sought from the patients before the data collection procedures.

### 2.2. Data Collection Procedures

#### 2.2.1. Inclusion and Exclusion Criteria

**Figure 1:**
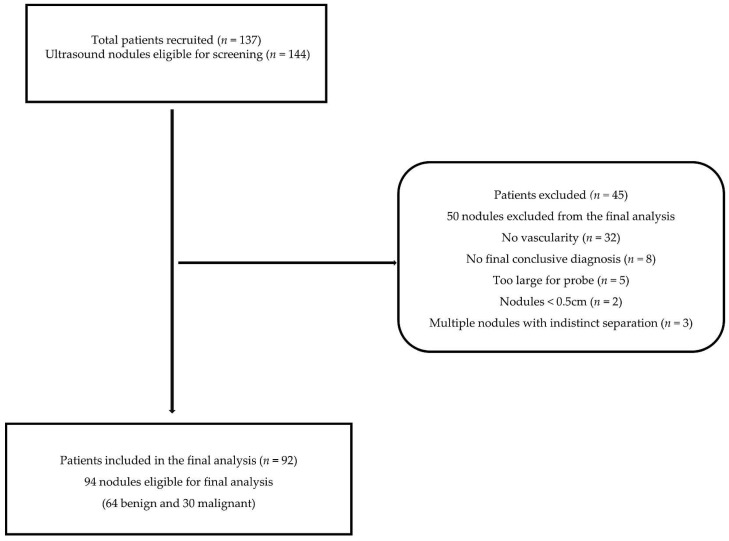
Representation of the patient inclusion and exclusion criteria.

A total of 94 thyroid nodule images (30 malignant; 64 benign), from 92 patients met the inclusion criteria of the present study (Figure 1). The inclusion criteria in this study required patients to all be consenting adults (≥18 years old) who had thyroid nodular disease or the suspicion of thyroid cancer and were scheduled for biopsy and/or subsequent thyroidectomy. Nodules that were ≥ 5mm were included in the study. For patients with multiple thyroid nodules, either the nodule with the most suspicious sonographic features (hypoechoic, microcalcifications, irregular margins, tall-than-wide, etc.) or if there were no obvious suspicious features, then the largest nodules on each lobe or the one/s for which biopsy and/or surgery was recommended were included in the study. The exclusion criteria necessitated patients who did not have a conclusive diagnosis as determined from either cytology results, histopathology results, or both, multinodular goitres without clearly isolated nodules, and non-vascular nodules.

#### 2.2.2. Ultrasound Imaging Procedures

A sole investigator with over 3 years experience in thyroid ultrasound scanning conducted the thyroid ultrasound imaging of all patients. An Aixplorer ultrasound machine (Supersonic Imagine, Aix-en-Provence, France) equipped with a 7–10 MHz linear transducer was used to conduct grey scale ultrasound scans, colour Doppler, power Doppler, and AngioPLUS Doppler ultrasound scans. The ultrasound machine settings were standardized and the same ultrasound scanning settings for the thyroid study were maintained to ensure consistency. For the Doppler ultrasound modes, the standard settings were set with a medium wall filter at the lowest pulse repetition frequency where no aliasing was encountered and the highest colour gain without signal noise. The resultant settings used in the study were a velocity scale of 10 cm/s with a colour map of 5 for CFI and PDI and a velocity scale of 4 cm/s with a colour map of 4 for the AngioPLUS modes.

Standard ultrasound protocols were observed to conduct the thyroid scans. Each patient lay in the supine position with minimal extension of the neck and coupling gel was applied. With the face turned away from the side of interest, the transducer was placed on the exposed side of the neck area. A minimum of 3 images of each target thyroid nodule were acquired in grey scale ultrasound, colour Doppler, power Doppler, and both colour and power Doppler with AngioPLUS modes. In the grey scale ultrasound mode, the images were acquired when most features suggestive of malignancy or benignity were observed, whereas in the Doppler ultrasound modes, images were acquired where the nodule demonstrated abundant vascularity and stable Doppler ultrasound signals.

Two thyroid surgeons with extensive experience independently conducted the ultrasound-guided fine-needle aspiration cytology (FNAC) and later provided the cytological and/or histopathological diagnosis of the thyroid nodules. Nodules with FNAC results of Categories 3 and 4 criteria, i.e., atypia of undetermined significance (AUS)/follicular lesion of undetermined significance (FLUS) and follicular neoplasm (FN)/suspicion of follicular neoplasm (SFN), were considered equivocal in the present study.

#### 2.2.3. Grey Scale Ultrasound Feature Assessment

**Figure 2:**
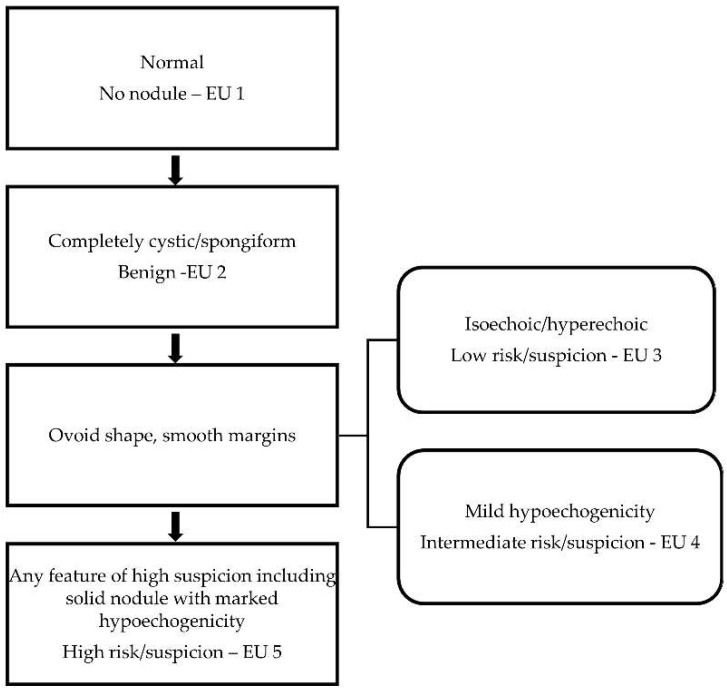
EU TIRADS malignancy risk categorization based on predictive ultrasound features.

The same investigator who conducted the imaging independently reviewed the thyroid nodule images and subjectively interpreted the ultrasound features based on echogenicity, composition, shape, margins, taller than wide ratio >1, and the presence/absence of calcifications. For the malignancy risk categorization, the interpretations were scored based on the EU TIRADS with the assistance of an online calculator for computation (www.gap.pe.kr/thyroidnodule.php (accessed on 17 October 2021)). Like most TIRADS, the common features that are indicative of high malignancy suspicion in EU TIRADS are irregular margins, marked hypoechogenicity, taller than wide shape, and microcalcifications [25]. However, for the classification of the high-risk/suspicion category, EU TIRADS requires just the presence of any of the common suspicious features or marked hypoechogenicity in a solid nodule [10,26]. The categorisation criteria for the EU TIRADS are shown in Figure 2. The cut-off point for malignancy risk stratification using sole grey scale ultrasound assessment was Category 5 (high risk/suspicion) in the present study. We hypothesized that central vascularization as an additional suspicious ultrasound feature could potentially improve the overall diagnostic accuracy of grey scale ultrasound assessment from the level of indeterminate ultrasound suspicion. Therefore, a cut-off point of Category ≥4 was used for the combined assessment of EU TIRADS with the Doppler modes.

#### 2.2.4. Doppler Ultrasound Feature Assessment

The vascularity features of the nodules were assessed using CFI and PDI and both modes coupled with AngioPLUS based on qualitative and quantitative approaches.

##### Qualitative Vascularity Assessment

**Figure 3:**
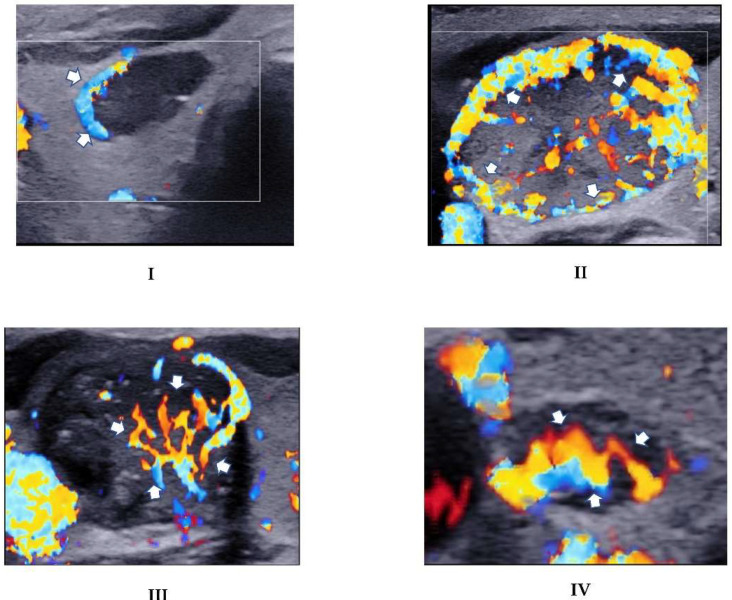
CFI images demonstrating the adopted qualitative vascularity grading. (**I**): Exclusive peripheral vascularity (arrows) in a nodule that had a conclusive benign histopathology diagnosis in a 61-year-old male patient. (**II**): Predominant peripheral vascularity demonstrated as more abundant vascularity in the outer regions (arrows) than the central regions in a histopathologically-benign nodule in a 53-year-old female patient. (**III**): Predominant vascularity in the central portion of a nodule (arrows) in a 44-year-old female who was diagnosed with PTC. (**IV**): Exclusive marked central flow (arrows) in a nodule diagnosed as PTC in a 51-year-old female.

Each nodule was subjectively evaluated for vascularity for all 4 Doppler modes (i.e., 1. CFI, 2. AngioPLUS + CFI (ACFI), 3. PDI, 4. AngioPLUS + PDI (APDI)). The subjective assessment was based on the qualitative grading criteria adapted from Chammas et al. [27]: Category I = exclusively peripheral vascularity; Category II = predominantly peripheral vascularity; Category III = predominant central vascularity, and Category IV = exclusively central blood flow. Two sets of interpretations of the qualitative vascularity grading with a one-month wash-out period between them were conducted for 40 images to assess intra-rater reliability. Nodules with Categories I and II features were considered suspicious for benignity, while those falling in Categories III and IV were considered suspicious for malignancy (Figure 3). The Doppler mode that resulted in superior diagnostic performance was then compared and combined with the grey scale ultrasound assessment and the diagnostic performances were evaluated to determine diagnostic value.

##### Quantitative Assessment

**Figure 4:**
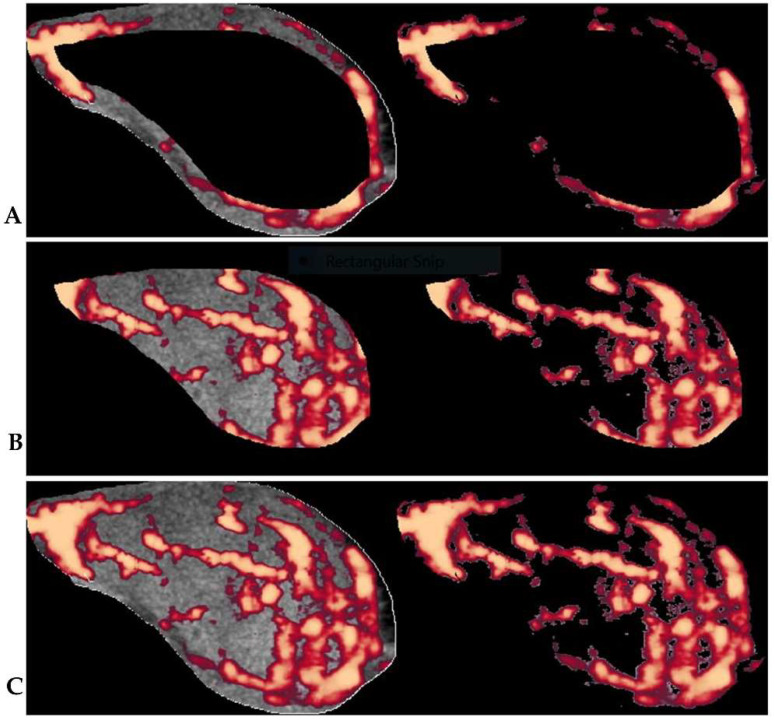
The illustration of the segmentation of thyroid nodule vascularity into: (**A**) peripheral and (**B**) central regions. (**C**) shows the primary ROI from which the overall vascularity is calculated. The VI for the different segments is calculated by the algorithm as the percentage between the total number of pixels (left) and the number of colour pixels without the grey scale pixels (right) within the segmented areas. The RVI is the ratio of the VIs of peripheral regions to that of central regions.

Three sets of thyroid nodule ultrasound images acquired for each Doppler mode (CFI, ACFI, PDI, and APDI) in the transverse planes were observed, documented, and saved for offline analysis. Microsoft Paint was used to manually outline regions of interest (ROI) and the images were saved in TIFF format and processed further in MATLAB (version 9.4.0.813654 R2018a; The Math Works, Natick, MA, USA). An image processing algorithm based on an offsetting principle for the regional vascularity segmentation in Doppler images, which was previously established by our research group [28,29,30], was used for the quantitative vascularity evaluation in the present study. The protocol and offset of 22%, which our research group established as diagnostically optimal for thyroid nodule regional vascularity segmentation [30], was used to delineate peripheral and central nodule regions. The central region was represented by the secondary ROI, which was extracted from the primary ROI (whole nodule) at the 22% offset, while the peripheral region was represented by the remaining outer segment of the primary ROI. Figure 4 illustrates the algorithm’s segmentation of central and peripheral vascularity regions in a thyroid nodule. Vascularity indices (VI) within peripheral and central thyroid nodule regions were determined from the averages of the three sets of readings for each of the Doppler modes. Furthermore, ratio analysis of the central and peripheral VIs was applied. A ratio vascularity index (RVI) of peripheral VI to central VI > 1 denoted predominant peripheral vascularity and ≤ 1 denoted predominant central vascularity. Based on the RVI method, the Doppler mode with the most optimal diagnostic performance was compared to the grey scale ultrasound and qualitative vascularity grading approaches and assessed in combination with the grey scale ultrasound assessment.

#### 2.2.5. Data Analysis and Statistical Analysis

Continuous data were classified as means +/− standard deviation, whereas categorical and/or nominal data were expressed as frequencies and percentages. The Shapiro–Wilk test was used to check the normality of the data. The Chi-square test was used to compare the differences in nodule classification data and peripheral and central vascularity index ratios of benign and malignant nodules. The paired samples T-test was used for testing the differences in the mean central and peripheral VI quantification between the CFI vs. ACFI and PDI vs. APDI modes. The Cohen’s kappa statistic (κ) complemented by the proportion agreement test was used for the intra-rater reliability assessment of qualitative grading of nodule vascularity using different Doppler modes. The sensitivity (SEN), specificity (SPEC), positive predictive values (PPV), negative predictive values (NPV), and area under the receiver operating characteristic curve (AUROCs) and their corresponding 95% confidence intervals (C.I.) were calculated with reference to the final cytology or pathology results. For the combined assessment of EU TIRADS and vascularity assessments, a nodule was predicted to be malignant if it met the cut-off criteria of both EU TIRADS (≤4) and that of either of the vascularity assessment approaches. The McNemar and Cochran Q’s tests were used for the comparative analysis of sensitivity and specificity, whereas the z-test was used to compare the different AUROCs. Multi-comparison testing was not applied to employ a more conservative approach and limit the false-negative rate (type 2 error) [31]. The tests were two-sided and *p* < 0.05 denoted statistical significance.

## 3. Results

### 3.1. Demographic Data

The mean age of all 92 patients (78 females; 14 males) was 53 ± 12.8 years (range: 21 to 75). On average, male patients were statistically significantly older than female patients (60.7 ± 9.5, range 44 to 71, vs. 51.7 ± 12.9, range 21 to 75, *p* = 0.01). However, there was no statistically significant difference in age between patients with malignant and benign thyroid nodules (53.5 ± 12.7, range: 31 to 74, and 52.9 ± 13, range: 21 to 75, respectively, *p* = 0.84). The most common histopathology diagnosis of the malignant nodules was papillary thyroid carcinoma (PTC, *n* = 26), while the remaining nodules were classified as follicular thyroid carcinoma (FTC, *n* = 3), and non-invasive follicular thyroid neoplasm with papillary-like nuclear features (NIFTP, *n* = 1). Among the 94 nodules, there were 40 nodules (31 benign; 9 malignant) with equivocal cytology. Two of the malignant nodules with equivocal cytology were FTCs while the rest were PTCs.

### 3.2. Thyroid Nodule Vascularity Assessments

The intra-rater agreement of using the qualitative method in the grading thyroid nodule vascularity was substantial (>0.6) with all the Doppler modes (Supplementary Table S1). The quantitative ratio analysis of regional vascularity computed as an RVI showed that for all the Doppler modes benign nodules presented with statistically significant predominant peripheral vascularity (RVI > 1, *p* < 0.01) rather than central vascularity, whereas there were no statistically significant differences between predominantly peripheral and predominantly central vascularity in malignant nodules (Supplementary Table S2).

The vascularity distributions in the peripheral and central nodule regions of conventional CFI and PDI Doppler modes were compared to those of the AngioPLUS modes, ACFI and APDI. The paired T-test results demonstrated that the addition of AngioPLUS to the conventional modes detected more vascularity as evidenced by the statistically significantly higher mean VIs in both segmented regions (peripheral VI: CFI vs. ACFI, t(93) = −8.89 and PDI vs. APDI, t(93) = −18.46; central VI: CFI vs. ACFI, t(93) = −7.64 and PDI vs. APDI, t(93) = −11.89, all *p* < 0.001). The results are shown in Table 1.

### 3.3. Diagnostic Performance Evaluation of EU TIRADS and Doppler Modes in Thyroid Nodule Malignancy Risk Stratification

#### 3.3.1. Sole Diagnostic Performance Assessments

Grey scale ultrasound assessment using EU TIRADS resulted in high sensitivity and lower specificity in stratifying all nodules and cytologically-equivocal nodules. The results are shown in Table 2.

The sole vascularity assessment of all nodules demonstrated that AngioPLUS significantly improved the sensitivity of conventional CFI (from 53.3% to 80%, *p* < 0.05) and PDI modes (from 46.7% to 83.3%, *p* < 0.05) and maintained comparable high specificities based on the qualitative assessment (Table 3). Similarly, for cytologically-equivocal nodules, the addition of AngioPLUS resulted in perfect sensitivity from that of PDI alone (100% from 66.7%, *p* < 0.05).

Quantitative vascularity assessment maintained high specificity and comparably lower sensitivity across all Doppler modes for stratifying all nodules and cytologically-equivocal nodules. The results are shown in Table 4. The addition of AngioPLUS to PDI (APDI) resulted in the highest sensitivity overall; however, this was only statistically significant to that of PDI in the assessment of cytologically-equivocal nodules (all: 66.7%; equivocal: 77.8%). Therefore, the addition of AngioPLUS to PDI based on qualitative vascularity assessment resulted in the most optimal sensitivity and specificity for stratifying all nodules and cytologically-equivocal nodules.

#### 3.3.2. Combination Approach Diagnostic Performance Assessments

**Figure 5:**
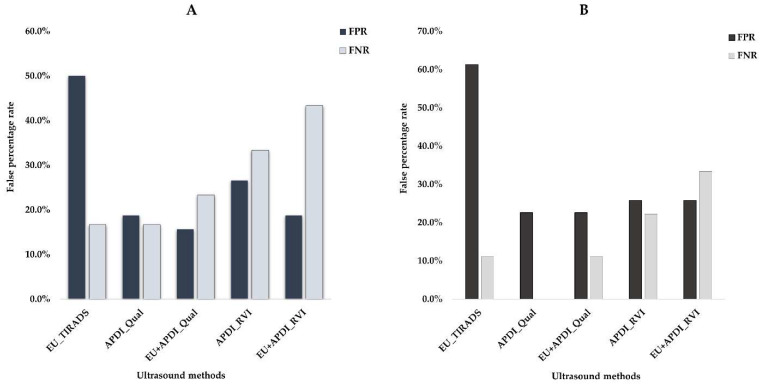
An illustration of the false-positive rates (FPR) and false-negative rates (FNR) for sole EU TIRADS and AngioPLUS + Power Doppler Imaging (APDI) based on qualitative (Qual) and quantitative ratio vascularity index (RVI) assessments, and combination approaches for stratifying: (**A**)—all nodules and (**B**)—cytologically-equivocal nodules.

Vascularity assessment based on AngioPLUS + Power Doppler Imaging (APDI) was combined with the EU TIRADS grey scale assessment to ascertain the diagnostic performance of the combination approaches in stratifying thyroid nodules. The results are demonstrated in Table 5. In the assessment of all nodules, the combination of APDI based on qualitative vascularity grading with EU TIRADS significantly improved the specificity of sole EU TIRADS (84.4% vs. 50%, *p* < 0.05) with a sensitivity that was insignificantly lower than that of sole EU TIRADS (76.7% vs. 83.3, *p* > 0.05). The false-negative rate (FNR) was best using the sole EU TIRADS (16.3%), while the combination with APDI resulted in a slight increase (23.3%). The addition of ADPI to EU TIRADS reduced the false positive rate (FPR) from the highest achieved with the sole EU TIRADS (15.6% vs. 50%). Figure 5A demonstrates the FPR and FPR results for stratifying all nodules. For stratifying cytologically-equivocal nodules, the addition of APDI to EU TIRADS resulted in the most optimal diagnostic performance. The sensitivity of the combination approach was comparable to the sole EU approach (88.9%), while the specificity was significantly improved compared to that of sole EU TIRADS (77.4% vs. 38.7%, *p* < 0.05). The combination approach maintained an FNR comparable to that of sole EU TIRADS (11.1%), and also achieved the lowest FPR (22.6%), while EU TIRADS had the highest FPR (61.3%). Figure 5B shows the FNR and FPR for cytologically-equivocal nodules. Overall, for the stratification of all nodules and cytologically-equivocal nodules, the addition of qualitatively assessed APDI to EU TIRADS had a higher discrimination ability than sole EU TIRADS.

The combination approach based on the addition of quantitatively assessed APDI to EU TIRADS resulted in high specificity, the lowest sensitivity, and the highest FNR for stratifying all nodules and cytologically-equivocal nodules.

## 4. Discussion

The present study evaluated the diagnostic value of AngioPLUS Doppler ultrasound for thyroid nodule malignancy-risk stratification based on qualitative vascularity grading and quantitative regional vascularity assessments in combination with EU TIRADS.

### 4.1. Grey Scale Ultrasound Assessment with EU TIRADS

In the present study, sole EU TIRADS had an optimal diagnostic performance with high sensitivity, low FNR, and low specificity for the malignancy-risk stratification of thyroid nodules. The high sensitivity with lower specificity of the EU TIRADS can be attributed to its criteria of the presence of any single predictive feature to denote high malignancy risk/suspicion. The diagnostic performance of EU TIRADS has been explored extensively in overall thyroid nodule assessment [32,33,34], but assessment of cytologically-equivocal nodules is scant. While the present study showed high sensitivity and low specificity in stratifying cytologically-equivocal nodules, some recent studies demonstrated a lower sensitivity (<55%) and higher specificity (≥70%) in discriminating FLUS/AUS and SFN/HC nodules [35,36]. The differences in the findings can be attributed to the smaller sample size, the use of computer-assisted EU TIRADS stratification, lack of subgrouping of the equivocal nodules, and the prevalence of PTCs in the present study. The present study’s findings can therefore be inferred as applicable to PTCs.

### 4.2. Qualitative Vascularity Assessment of AngioPLUS Combined with EU TIRADS

The present study demonstrated that coupling AngioPLUS with conventional Doppler modes resulted in a more optimal diagnostic performance than conventional Doppler modes alone. The superior detection of microvascular flow with AngioPLUS resulted in high specificity and high sensitivity, which was comparable to that of EU TIRADS alone. A predominantly central vascularity pattern has previously been suggested as an independent thyroid nodule malignancy risk factor [27]; however, its diagnostic performance with conventional CFI and DPI resulted in ambiguous findings [18,37]. Our findings of high specificity and low sensitivity with conventional Doppler modes using this vascularity pattern concur with those of previous studies [38,39]. However, some studies have shown variable findings of either high sensitivity or high sensitivity and specificity [40,41]. Rosario et al. [42] demonstrated that there was no additional diagnostic value in combining PDI vascularity assessment with sole grey scale ultrasound assessment since the diagnostic performance outcomes of the combination approach remained comparable to those of the sole grey scale ultrasound (SEN: 88.7% vs. 89.4%; SPEC: 68.2% vs. 66.4%). The varying study designs and the diversity of ultrasound machines that influence the poor sensitivity of conventional Doppler modes in detecting microvascular flow contribute to the different findings.

In the present study, the combination of qualitatively graded APDI with EU TIRADS improved the overall diagnostic efficacy of sole EU TIRADS for stratifying all nodules. Although sole EU TIRADS had the highest sensitivity and lowest FNR overall, the highest FPR is suggestive of a high unnecessary biopsy rate with sole use. The combination of EU TIRADS with APDI with a lower FPR could have reduced the unnecessary biopsy rate by 34.4% while sufficiently detecting true malignant cases as it maintained a comparably high sensitivity. The slight increase in FNR with the combination approach would have resulted in only two cases of true malignancies being missed. Furthermore, the combination of APDI with EU TIRADS proved to be best for stratifying cytologically-equivocal nodules over sole EU TIRADS. By maintaining a high sensitivity while significantly increasing the specificity and resulting in low FNR and FPR compared to sole EU TIRADS, the combination approach had high discriminating ability and could have lowered the unnecessary biopsy rate of EU TIRADS by 38.7%. Based on our results, we can posit that AngioPLUS has additional diagnostic value in the differentiation of thyroid nodules, as it optimises both the sensitivity and specificity and therefore can potentially limit unnecessary biopsy rates. Combined with EU TIRADS at the cut-off point of intermediate suspicion category, the false-positive rate is lowered compared to sole EU TIRADS using a high suspicion category cut-off point. This combination approach could potentially be used to follow up cytologically equivocal thyroid nodules. Integrating it into the routine diagnostic workflow may result in the conservative diagnosis of thyroid nodules by the optimal detection of cancers and limited unnecessary repeated biopsies.

Our findings of optimal sensitivity and specificity with AngioPLUS compared to sole grey scale assessment concur with studies of thyroid nodule assessment based on SMI. A previous study reported an improved sensitivity of PDI using SMI (41.8% to 75.9%) and an excellent diagnostic efficacy (AUROC: 0.92) in combination with grey scale ultrasound features [43]. In another study, SMI combined with ACR TIRADS improved the sensitivity, specificity, and AUROC of sole ACR TIRADS (SEN: 65.1% to 93.8%; SPEC: 93% to 94.4%; AUROC: 0.88 to 0.95) [44]. Although the combination approaches in both studies showed an improved overall diagnostic efficacy, the different diagnostic outcomes of the two sole TIRADS may be explained by the different malignancy risk stratification criteria. Our study used the EU TIRADS at the high malignancy risk category based on a pattern-based approach, whereas the aforementioned study used ACR at the moderate risk category based on a score-based approach. Contrastingly, in other studies SMI combined with grey scale ultrasound feature assessment failed to significantly improve the diagnostic performance [45,46]. The variable findings can be attributed to different TIRADS, SMI assessment using the monochromatic mode, and vascularity grading, where intranodular vascularity was classified as mild or extensive vascularity, or both.

### 4.3. Quantitative Vascularity Assessment of AngioPLUS in Combination with EU TIRADS

In the present study, the quantitative vascularity assessment approach demonstrated higher VIs of central and peripheral vascularity regions with the AngioPLUS modes than the sole conventional Doppler modes. Therefore, these results substantiate the increased sensitivity in microvascularity detection in thyroid nodules with AngioPLUS than with conventional Doppler modes. The regional vascularity ratio analysis based on an RVI > 1 at an offset of 22% affirmed that predominant peripheral vascularity is prevalent in benign nodules. However, the approach had high specificity and lower sensitivity even with AngioPLUS and in combination with EU TIRADS for the overall assessment of all nodules. Quantitative vascularity assessment approaches had been evaluated in a few previous studies mainly using conventional Doppler modes. A recent study based on the quantification of SMI reported that higher SMI pixel counts in malignant thyroid nodules may help differentiate them from benign nodules [47]. However, that approach also had low sensitivity (40.5%) and high specificity (91.3%). Yoon et al. [48] reported a low diagnostic performance of PDI using sole VI assessment with a significant reduction in specificity from that of grey scale ultrasound alone using the combination approach. Contrarily, in another study based on a 90% central to 10% peripheral ratio for the regional segmentation of intranodular vascularity, the overall VI, central VI, and peripheral VI for vascularity densities yielded high sensitivity and low specificity [49]. However, neither peripheral nor central vascularity was predominant in benign or malignant nodules in that study. Contrary to the present study, the only study that compared sole qualitative and quantitative vascularity assessment reported a higher sensitivity using quantitative central vascular area, whereas the specificity was comparable (SEN: 90% vs. 67.5%; SPEC: 88.1% vs. 88%) [50]. The differences in the methodologies of determining central vascularity may explain the variable findings.

In the present study, the APDI mode resulted in a more optimal sensitivity, which was lower than that of sole EU TIRADS, balanced with higher specificity and NPV, which were maintained in the combination approach with EU TIRADS. For the assessment of cytologically-equivocal thyroid nodules, APDI had the most optimal diagnostic performance; however, the combination approach with EU TIRADS resulted in reduced sensitivity and sustained specificity. Although there is potential to reduce the unnecessary biopsy rates with this approach, the large sacrifice of the sensitivity would be a drawback in clinical application considerations. This is because the reduced sensitivity increases the false-negative rate, which may delay treatment of thyroid cancer patients. Ultimately, the ratio analysis approach of RVI > 1 may accurately stratify benign nodules but may not be ideal for ruling in disease. Therefore, qualitatively graded APDI in combination with EU TIRADS is more optimal for best stratifying cytologically-equivocal thyroid nodules.

### 4.4. Limitations

The sample size was small and most malignant nodules were PTCs, thereby limiting the generalisability of the findings to other cancers. The category-specific diagnostic performance evaluation of AngioPLUS Doppler ultrasound in cytologically-equivocal nodules could not be conducted. Due to the selection of patients with FNAC and/or histopathology results, we cannot exclude selection bias. However, due to the limited diagnostic performance evaluation of the AngioPLUS Doppler ultrasound, along with several TIRADS, our findings may guide larger multi-centre prospective validation studies with multiple raters and different types of thyroid cancers.

## 5. Conclusions

Qualitatively-graded APDI has additional diagnostic value in thyroid nodule differentiation as it can improve the diagnostic efficacy of EU TIRADS by optimising both the sensitivity and specificity. It has greater potential for improving the diagnosis of cytologically-equivocal nodules and limiting unnecessary biopsy rates. Quantitative vascularity assessment using APDI adequately discriminates benign nodules but is not effective for ruling in malignancy even when combined with EU TIRADS.

## Figures and Tables

**Table 1:** Paired comparison of thyroid nodule vascularity detection between conventional Doppler modes and AngioPLUS modes.

Doppler Mode Pairs at Segmented Regions		Descriptive Statistics			Paired Sample *t*-Tests		
		Mean VI	SD	SEM	t	df	*p*-Value (2-Tailed)
**Peripheral VI**	CFI	44.92	16.05	1.66	−8.89	93	<0.001
	CFI + AngioPLUS	55.29	16.74	1.73			
	PDI	30.91	12.81	1.32	−18.46	93	<0.001
	PDI + AngioPLUS	57.34	17.82	1.84			
Central VI	CFI	36.70	19.88	2.05	−7.64	93	<0.001
	CFI + AngioPLUS	48.07	19.20	1.98			
	PDI	26.14	14.76	1.52	−11.89	93	<0.001
	PDI + AngioPLUS	49.55	21.29	2.20			

CFI = colour flow imaging, PDI = power Doppler imaging, SD = standard deviation, SEM = standard error of mean, df = degrees of freedom, VI = vascularity index.

**Table 2:** Diagnostic performance of EU TIRADS for the stratification of all nodules and cytologically-equivocal nodules.

	EU TIRADS	
Diagnostic Performance Measures	All Nodules (*n* = 94)	Equivocal Nodules (*n* = 40)
SEN (%)	83.3 (65.3; 94.4)	88.9 (51.8; 99.7)
SPEC (%)	50.0 (37.2; 62.8)	38.7 (21.8; 57.8)
PPV (%)	43.9 (30.7; 57.6)	29.6 (13.8; 50.2)
NPV (%)	86.5 (71.2; 95.5)	92.3 (64.0; 99.8)
AUROC	0.67 (0.57; 0.76)	0.62 (0.47; 0.78)

SEN = sensitivity, SPEC = specificity, PPV = positive predictive value, NPV = negative predictive value,. AUROC = area under the receiver operating characteristic curve.

**Table 3:** Diagnostic performance of qualitative vascularity grading in thyroid nodule risk-stratification of all nodules and cytologically-equivocal nodules.

		Qualitative Vascularity Grading Modes			
Nodules	Diagnostic Performance	CFI	ACFI	PDI	APDI
**All** **(*n*** ** = 94)**	SEN (%)	53.3 * (34.3; 71.7)	80.0 (61.4; 92.3)	46.7 ^‡^ (28.3; 65.7)	83.3(65.3; 94.4)
	SPEC (%)	92.2 (82.7; 97.4)	82.8 (71.3; 91.1)	95.3 (86.9; 99.0)	81.3 (69.5; 89.9)
	NPV (%)	80.8 (69.9; 89.1)	89.8 (79.2; 96.2)	79.2 (68.5; 87.6)	91.2 (80.7; 97.1)
	PPV (%)	76.2 (52.8; 91.8)	68.6 (50.7; 83.1)	82.4 (56.6; 96.2)	67.6 (50.2; 82.0)
	AUROC	0.73 (0.63; 0.82)	0.81 (0.73; 0.90)	0.71 ^‡^ (0.62; 0.80)	0.82(0.74; 0.91)
**Equivocal** **(*n*** ** = 40)**	SEN (%)	66.7 * (29.9; 92.5)	88.9 (51.8; 99.7)	66.7 ^‡^ (29.9; 92.5)	100 (66.4; 100)
	SPEC (%)	90.3 (74.2; 98.0)	80.6 (62.5; 92.5)	93.5 (78.6; 99.2)	77.4 (58.9; 90.4)
	NPV (%)	90.3 (74.2; 98.0)	96.2 (80.4; 99.9)	90.6 (75.0; 98.0)	100 (.)
	PPV (%)	66.7 (29.9; 92.5)	57.1 (28.9; 82.3)	75.0 (34.9; 96.8)	56.3 (29.2; 80.2)
	AUROC	0.79 (0.61; 0.96)	0.85 (0.72; 0.98)	0.80 (0.63; 0.97)	0.89 (0.81; 0.96)

SEN = sensitivity, SPEC = specificity, PPV = positive predictive value, NPV = negative predictive value, AUROC = area under the receiver operating characteristic curve, CFI = colour flow imaging, ACFI = AngioPLUS + CFI, PDI = power Doppler imaging, APDI = AngioPLUS + PDI, (.) = value dependent on disease prevalence. * = *p* < 0.05 relative to ACFI, ^‡^ = *p* < 0.05 relative to APDI.

**Table 4:** Diagnostic performance of quantitative vascularity grading (RVI ≥ 1) in thyroid nodule risk- stratification of all nodules and cytologically-equivocal nodules.

		Quantitative Vascularity Grading Modes			
Nodules	Diagnostic Performance	CFI	ACFI	PDI	APDI
**All** **(*n*** ** = 94)**	SEN (%)	56.7 (37.4 ; 74.5)	60.0 (40.6 ; 77.3)	46.7 (28.3 ; 65.7)	66.7 (47.2 ; 82.7)
	SPEC (%)	81.3 (69.5 ; 89.9)	73.4 (60.9 ; 83.7)	65.6 (52.7 ; 77.1) ^§§^	73.4 (60.9 ; 83.7)
	NPV (%)	80.0 (68.2 ; 88.9)	79.7 (67.2 ; 89.0)	72.4 (59.1 ; 83.3)	82.5 (70.1 ; 91.3)
	PPV (%)	58.6 (38.9 ; 76.5)	51.4 (34.0 ; 68.6)	38.9 (23.1 ; 56.5)	54.1 (36.9 ; 70.5)
	AUROC	0.69 (0.59 ; 0.79) ^††^	0.67 (0.56 ; 0.77)	0.56 (0.45 ; 0.67)	0.70 (0.60 ; 0.80) ^††^
**Equivocal** **(*n*** ** = 40)**	SEN (%)	66.7 (29.9 ; 92.5)	55.6 (21.2 ; 86.3)	33.3 (7.5 ; 70.1) ᵻ	77.8 (40.0 ; 97.2) ᵻ
	SPEC (%)	83.9 (66.3 ; 94.5)	67.7 (48.6 ; 83.3)	61.3 (42.2 ; 78.2)	74.2 (55.4 ; 88.1)
	NPV (%)	89.7 (72.6 ; 97.8)	84.0 (63.9 ; 95.5)	76.0 (54.9 ; 90.6)	92.0 (74.0 ; 99.0)
	PPV (%)	54.5 (23.4 ; 83.3)	33.3 (11.8 ; 61.6)	20.0 (4.3 ; 48.1)	46.7 (21.3 ; 73.4)
	AUROC	0.75 (0.58; 0.93) ^††^	0.62 (0.43; 0.81)	0.47 (0.29; 0.66)	0.76 (0.60; 0.92) ^††^

SEN = sensitivity, SPEC = specificity, PPV = positive predictive value, NPV = negative predictive value, AUROC = area under the receiver operating characteristic curve, RVI = ratio vascularity index, CFI = colour flow imaging, ACFI = AngioPLUS + CFI, PDI = power Doppler imaging, APDI = AngioPLUS + PDI,. ^§§^ = *p* < 0.01 with reference to CFI; ᵻ = *p* < 0.05 with reference to all other modes, ^††^ = *p* < 0.01 with reference to PDI.

**Table 5:** Diagnostic performance assessment of EU TIRADS in combination with qualitative grading and quantitative vascularity assessment in thyroid nodule risk-stratification.

Nodules	Diagnostic Performance Measures	GSU	GSU + Qualitative Vascularity	GSU + Quantitative Vascularity
		EU	EU + APDI_Qual	EU + APDI_RVI
**All** **(*n* = 94)**	SEN (%)	83.3 (65.3 ; 94.4)	76.7 (57.7 ; 90.1)	58.1 (39.1 ; 75.5) *
	SPEC (%)	50.0 (37.2 ; 62.8)	84.4 (73.1 ; 92.2) ***	81.0 (69.1 ; 89.8) ***
	PPV (%)	43.9 (30.7 ; 57.6)	69.7 (51.3 ; 84.4)	60.0 (40.6 ; 77.3)
	NPV (%)	86.5 (71.2 ; 95.5)	88.5 (77.8 ; 95.3)	79.7 (67.8 ; 88.7)
	AUROC	0.67 (0.57 ; 0.76)	0.81 (0.72 ; 0.89) *	0.70 (0.60 ; 0.80)
**Equivocal** **(*n* = 40)**	SEN (%)	88.9 (51.8 ; 99.7)	88.9 (51.8 ; 99.7)	66.7 (29.9 ; 92.5)
	SPEC (%)	38.7 (21.8 ; 57.8)	77.4 (58.9 ; 90.4) ***	74.2 (55.4 ; 88.1) ***
	PPV (%)	29.6 (13.8 ; 50.2)	53.3 (26.6 ; 78.7)	42.9 (17.7 ; 71.1)
	NPV (%)	92.3 (64.0 ; 99.8)	96.0 (79.6 ; 99.9)	88.5 (69.8 ; 97.6)
	AUROC	0.62 (0.47; 0.78)	0.83 (0.70; 0.96) ***	0.70 (0.52; 0.89)

SEN = sensitivity, SPEC = specificity, PPV = positive predictive value, NPV = negative predictive value, AUROC = area under the receiver operating characteristic curve, RVI = ratio vascularity index, GSU = grey scale ultrasound, EU = European TIRADS, APDI = AngioPLUS + power Doppler imaging, Qual = qualitative vascularity grading, * = *p* < 0.05 with reference to EU, *** = *p* < 0.001 with reference to EU.

## Data Availability

The clinical and ultrasound data are publicly unavailable due to privacy protection and patient confidentiality reasons.

## Supplementary Materials

The following are available online at https://www.mdpi.com/article/10.3390/biomedicines10071554/s1, Table S1: Intra-rater agreement of qualitative thyroid nodule vascularity assessment using different Doppler modes, Table S2: Distribution of predominantly peripheral (RVI > 1) and predominantly central vascularity (RVI ≤ 1) in benign and malignant thyroid nodules for different Doppler modes.
