# Supplementary material for: Diagnostic Value of AngioPLUS Microvascular Imaging in Thyroid Nodule Diagnosis Using Quantitative and Qualitative Vascularity Grading
Source: Biomedicines. 2022 Jun 29;10(7):1554. doi: 10.3390/biomedicines10071554 (PMC9313421; doi:10.3390/biomedicines10071554)
Supplement: Supplementary file 1 [file biomedicines-10-01554-s001.zip › biomedicines-1774131-supplementary.pdf]

Supplementary Tables

Supplementary Table S1. Intra-rater agreement of qualitative thyroid nodule vascularity assessment using different Doppler modes.

Vascularity Mode	Kappa rate of agreement (κ)	Proportions agreement (%)
**CFI**	0.75	80
**PDI**	0.64	77
**ACFI**	0.76	80
**APDI**	0.71	77

CFI = colour flow imaging, PDI = power Doppler imaging, ACFI = AngioPLUS +CFI, APDI = AngioPLUS + PDI

Supplementary Table S2. Distribution of predominantly peripheral (RVI > 1) and predominantly central vascularity (RVI ≤1) in benign and malignant thyroid nodules for different Doppler modes.

**Nodule category**	**Nodule Status**	**Doppler Modes**							
		**CFI**		**PDI**		**ACFI**		**APDI**	
		**RVI >1**	**RVI ≤1**	**RVI >1**	**RVI ≤1**	**RVI >1**	**RVI ≤1**	**RVI >1**	**RVI ≤1**
All	B (n = 64)	52^{***}	12	42^{**}	22	47^{***}	17	52^{***}	12
	M (n = 30)	13	17	16	14	12	18	10	20
Equivocal	B (n = 31)	26^{***}	5	19	12	21^{**}	10	23^{***}	8
	M (n = 9)	3	6	6	3	4	5	2	7

CFI = colour flow imaging, PDI = power Doppler imaging, ACFI = AngioPLUS +CFI, APDI = AngioPLUS + PDI , B = benign; M = malignant; $^{*}= p < 0.05$, $^{**}= p < 0.01$, $^{***}= p < 0.001$, *RVI = ratio vascularity index,*
